# Protein *O*‐glycosylation in the Bacteroidota phylum

**DOI:** 10.1002/2211-5463.70041

**Published:** 2025-04-15

**Authors:** Lonneke Hoffmanns, Dennis Svedberg, André Mateus

**Affiliations:** ^1^ Department of Chemistry Umeå University Umeå Sweden; ^2^ The Laboratory for Molecular Infection Medicine Sweden (MIMS) Umeå University Umeå Sweden; ^3^ Umeå Center for Microbial Research (UCMR) Umeå University Umeå Sweden

**Keywords:** Bacteroidota, glycosylation, glycosyltransferase, Gram‐negative bacteria, microbiome

## Abstract

Glycans play crucial roles in bacteria, such as providing structural integrity or enabling interactions with the ecosystem. They can be linked to lipids, peptides, or proteins. In proteins, they modify either asparagine (*N‐*glycosylation) or serine or threonine (*O‐*glycosylation). Species of the Bacteroidota phylum, a major component of the human microbiome and marine and soil ecosystems, have a unique type of *O‐*glycosylation that modifies multiple noncytoplasmic proteins containing a specific amino acid sequence. Only a small number of species have currently been characterized, but within one species, generally all proteins are modified with the same glycan structure. Most species share a common inner part but differ in the sugar composition and branching of the outer part of their glycan. This suggests that the biosynthesis of the glycan occurs in two separate steps. Both the inner core and the outer glycan are likely assembled from nucleotide‐activated monosaccharides on undecaprenyl phosphate on the cytoplasmic side of the inner membrane, prior to being flipped to the periplasm and transferred to the protein. A genomic locus responsible for the biosynthesis of the outer glycan has been identified, containing some conserved genes across species. Despite substantial progress in the characterization of this *O*‐glycosylation system, its function, the overall diversity of glycan structures across the phylum, and the complete biosynthetic pathway remain mostly unknown. Due to the importance of this group of species for the human gut microbiome, elucidating these aspects can open up strategies to modulate the composition of the microbiome community toward a healthy state.

## Graphical abstract

Species of the Bacteroidota phylum exhibit a unique *O*‐glycosylation system. It modifies noncytoplasmic proteins on a specific amino acid motif with a shared glycan core but a species‐specific outer glycan. A locus of multiple glycosyltransferases responsible for the synthesis of the outer glycan has been identified. While progress has been made, the functions and diversity of the glycans remain unclear.

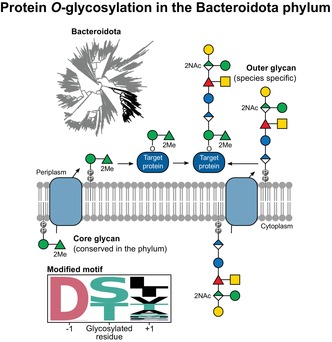

**Abbreviations**
GlcNAc: *N*‐acetylglucosamine
GT: glycosyltransferase
LFG: locus of fragilis glycosylation
LPS: lipopolysaccharide
MurNAc: *N*‐acetylmuramic acid
OMV: outer membrane vesicle
OTase: oligosaccharyl transferase
tRNA: transfer ribonucleic acid
Und‐P: undecaprenyl phosphate

Bacteria produce a wide variety of glycan structures playing important roles in various cellular processes [1]. Compared with eukaryotes, these contain unique bacterial monosaccharides and modifications of the sugar chains. Glycans are present in both Gram‐negative and Gram‐positive bacteria and can consist solely of carbohydrates or be linked to lipids, short peptides, or proteins. Many of these structures provide integrity to the cell, protection from host defenses, virulence, or surface adhesion [1]. Examples are as follows: the peptidoglycan (the cell wall), a structure made up of linear chains of alternating *N*‐acetylglucosamine (GlcNAc), and *N*‐acetylmuramic acid (MurNAc) residues connected by short peptide crosslinks [2]; teichoic acids, which are anionic glycopolymers of glycerol phosphate, ribitol phosphate, or sugar phosphate found in Gram‐positive bacteria covalently bound to the peptidoglycan or anchored to the outer leaflet of the cytoplasmic membrane [3]; lipopoly‐ and lipooligosaccharides in Gram‐negative bacteria, which consist of an outer membrane glycolipid called lipid A, a core oligosaccharide, and in the case of LPS an outer *O*‐antigen polysaccharide repeat [4]; exopolysaccharides and capsules, which are high molecular weight extracellular polysaccharides of varying composition depending on the species [5, 6]; and osmoregulated periplasmic glucans, which consist of a β‐glucan backbone that can be modified with different substituents [7]. Protein glycosylation is also present in bacteria—both *O*‐glycosylation, in which the modified amino acids are serine or threonine, and *N*‐glycosylation, in which the modified amino acid is asparagine (the latter only described in Gram‐negative bacteria) [8]. The biosynthesis and structure of these glycans can be quite different from eukaryotes.

This review focuses on protein glycosylation systems in bacteria, and in particular on a unique *O*‐glycosylation system present in species of the Bacteroidota phylum [9]. The function of this system remains unclear, but it plays a role in the survival of these species in their natural environment. This has important consequences for human health, since species of the Bacteroidota phylum are among the most abundant and prevalent species in human gut microbiomes [10].

## Bacterial protein glycosylation

In bacteria, four different protein glycosylation pathways have been identified, two types responsible for *N*‐glycosylation and two for *O*‐glycosylation.

The canonical *N*‐glycosylation pathway assembles the glycan from nucleotide‐activated monosaccharides stepwise onto undecaprenyl phosphate (Und‐P), after which the complete glycan is flipped across the inner membrane into the periplasm [1, 8]. This is followed by the transfer of the glycan onto asparagine residues within the D/E‐X1‐N‐X2‐S/T (where X1 and X2 cannot be proline) motif in different proteins.

The noncanonical bacterial *N*‐glycosylation pathway transfers monosaccharides from nucleotide‐activated precursors directly to the proteins in the cytoplasm [1, 8]. In contrast to eukaryotic *N*‐glycosylation, no conserved *N*‐glycan core structure has been observed in bacteria [11]. Protein *N*‐glycosylation has been shown to be important for bacterial survival, adhesion, autoaggregation, and pathogenicity in Gram‐negative bacteria, but has not been identified in Gram‐positive bacteria [12].

For *O*‐glycosylation, one pathway is oligosaccharyl transferase (OTase)‐independent and present in both Gram‐negative and ‐positive bacteria. This pathway uses nucleotide‐activated monosaccharides as a donor for soluble glycosyltransferases present in the cytoplasm, facilitating the stepwise addition of monosaccharides to the protein [1]. Its main function is *O*‐glycosylation of the flagella and adhesins (extracellular proteins) [13]. For flagellar *O*‐glycosylation, no amino acid recognition sequence beyond the modified S/T has been identified [13]. In adhesins, *O*‐glycosylation occurs in regions with serine‐ and threonine‐rich repeats [14]. The *O*‐glycans vary in structure from single monosaccharides to oligosaccharides of more than 10 sugars, and are essential for filament assembly, bacterial motility, adherence, and biofilm formation [13, 14].

The other type of *O*‐glycosylation is OTase‐dependent and mainly present in Gram‐negative bacteria [1]. This type of glycosylation does not occur in eukaryotes but is reminiscent of the eukaryotic *N*‐glycosylation pathway, the canonical *N*‐glycosylation pathway in bacteria and even parts of the LPS biosynthetic pathway in Gram‐negative bacteria [11]. Like the canonical *N*‐glycosylation pathway, it uses nucleotide‐activated monosaccharides and assembles the glycan first onto Und‐P on the cytosolic side prior to being flipped into the periplasm by a flippase. The glycan is then added to a serine or threonine on the target protein by an *O*‐OTase in the periplasm.

## The unique *O*‐glycosylation system of Bacteroidota

In contrast to the bacterial *N*‐glycosylation, *O*‐glycosylation generally does not occur on a specific sequence motif, and the glycan location is difficult to predict [14]. The only exception known so far is a unique type of *O*‐glycosylation in the Bacteroidota phylum. This type of bacterial *O*‐glycosylation was first shown in *F. meningosepticum*, in which it occurs on serine and threonine preceded by an aspartic acid and followed preferentially by hydrophobic amino acids [15, 16].

### Bacteroidota‐specific *O‐*glycosylation is driven by a sequence motif in noncytoplasmic proteins

**Fig. 1:**
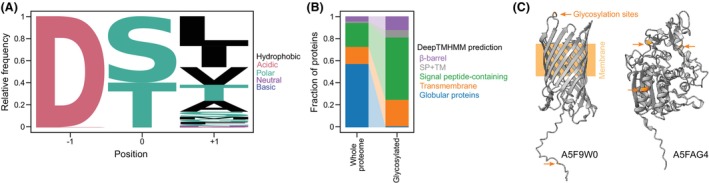
Unique *O‐*glycosylation system of Bacteroidota occurs at a specific motif in soluble regions of noncytoplasmic proteins. (A) Sequence preference of *O*‐glycosylated peptides from a reanalysis of previously acquired glycoproteomes [17, 18, 19, 20, 24] shows that the glycosylated amino acid (serine or threonine) is nearly always preceded by an aspartic acid residue and preferentially followed by a hydrophobic residue. The amino acids L/I/V/T/A/S together make up approximately 90% of the amino acids found downstream of the glycosylated residue, with M/F/C/G being uncommon at around 2% each and N/E/Q/P/D having less than 1% occurrence. (B) Glycosylated proteins are enriched in transmembrane and signal peptide‐containing proteins as predicted by DeepTMHMM [25], indicating that glycosylation only occurs in noncytoplasmic proteins. (C) Examples of measured glycosylated sites in soluble regions of two proteins of *Flavobacterium johnsoniae* [19]. For transmembrane proteins (A5F9W0) both periplasmic and extracellular motifs can be glycosylated. Proteins are referred to by their UniProt entry number, and glycosylated sites are based on their previous identification [19].

The sequence preference for the glycosylation motif has become clear after multiple glycoproteomics analyses (Fig. 1A). Aspartic acid almost always precedes the glycosylated residue, and substitution with the most similar amino acid (glutamic acid) is not tolerated in *B. fragilis* [9], but has been observed on rare occasions in other species [17, 18]. In all species, there is a slight preference for serine over threonine at the position that is glycosylated (Fig. 1A, Fig. S1a), and the amino acid that follows is not influenced by the identity of the glycosylated residue (Fig. S1c). Different species seem to have a preference for this last residue in the motif [19, 20], which is most commonly one of the hydrophobic residues leucine, isoleucine, valine, threonine, alanine, serine, or methionine, in this order. Glycine, phenylalanine, and cysteine have also occasionally been detected (Fig. S1b), but attempting to introduce glycine at this position by genetic manipulation of *B. fragilis* resulted in loss of glycosylation [9]. This type of *O*‐glycosylation appears to be abundant in Bacteroidota, since about half of the noncytoplasmic proteins contain the glycosylation motif and the serine or threonine in the motif is generally nearly fully substituted (at least when considering the more common motifs) [15, 18, 19, 20, 21, 22, 23, 24]. In addition, the introduction of an engineered glycosylation motif into a protein leads to its glycosylation [21]. It is even possible to cross‐glycosylate proteins by different species, since the introduction of *B. fragilis* proteins into *T. forsythia* results in glycosylation of those proteins with the *T. forsythia O*‐glycan and vice versa [23].

The *O*‐glycoproteins identified in different species of Bacteroidota are predominantly localized in the periplasm, and inner and outer membranes (Fig. 1B) [9, 17, 18, 19, 20, 21, 24, 26]. Most *O*‐glycosylation sites are located in soluble regions of proteins, with transmembrane proteins having glycosylated sites mostly facing the periplasm, but sporadically also the cell exterior (Fig. 1C) [17, 19, 24].

### *O‐*glycosylation is widespread in the Bacteroidota phylum

**Fig. 2:**
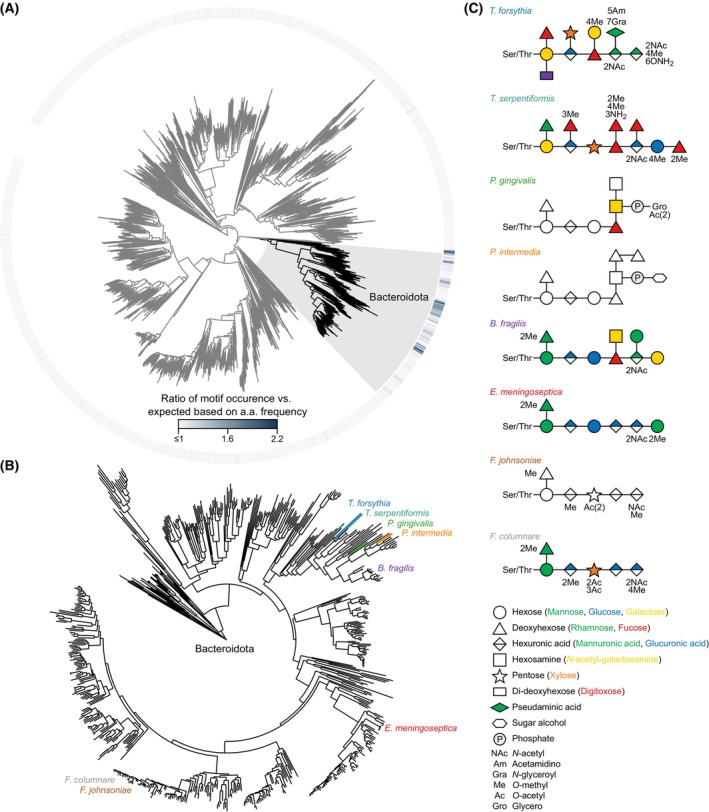
Phylogenetic distribution of currently characterized *O*‐glycans. (A) Phylogenetic tree of bacteria, including representative strains of each genus for each branch as defined by the Genome Taxonomy Database [31], and with the Bacteroidota phylum highlighted. The outer ring represents the ratio of the glycosylation motif D‐S/T‐A/L/V/I/M/T frequency in predicted noncytoplasmic proteins to that expected by chance, accounting for species‐specific amino acid frequencies. (B) Phylogenetic tree of Bacteroidota, including representative strains of each species with species for which the *O‐*glycoproteome, has been chemically characterized highlighted. (C) Chemical structure of characterized *O‐*glycans in different species [16, 17, 18, 19, 20, 21, 24, 27, 28, 29].

The glycosylation motif is enriched mainly in Bacteroidota when compared to all other bacterial species (Fig. 2A). It appears enriched in nearly all families within the phylum, but not in all species. This suggests that it evolved in an early common ancestor of this clade. Out of all the species of Bacteroidota, O‐glycans have only been partially or fully characterized for a handful of species sparsely spread across the phylum (Fig. 2B) [16, 17, 18, 19, 20, 21, 24, 27, 28, 29]. For most species, proteins are generally modified with the same glycoform, but this structure varies from species to species (Fig. 2C). For some species, there are slight variations of the glycoform that are present in lower abundance [19, 24]. There appears to be a common trisaccharide at the reducing end of the glycan consisting of a hexose (mannose), deoxyhexose (rhamnose), and hexuronic acid (glucuronic acid). The only exceptions to this are the investigated *Tannerella* species, but these species seem to be distinct from the other characterized species, since they predominantly glycosylate the two S‐layer proteins [18, 26]. *T. forsythia* is suggested to have several different abundant glycoforms that are specific for different glycosylation motifs [20], and some strains exchange the pseudaminic acid residue for its stereoisomer legionaminic acid [30]. *T. serpentiformis* has two different abundant glycoforms that show a preference for specific glycosylation sites on the protein [18]. The outer glycan shows structural diversity between species, but closely related species have more structurally similar outer glycans (Fig. 2C).

### Biosynthesis pathway of *O‐*glycans is conserved across species

**Fig. 3:**
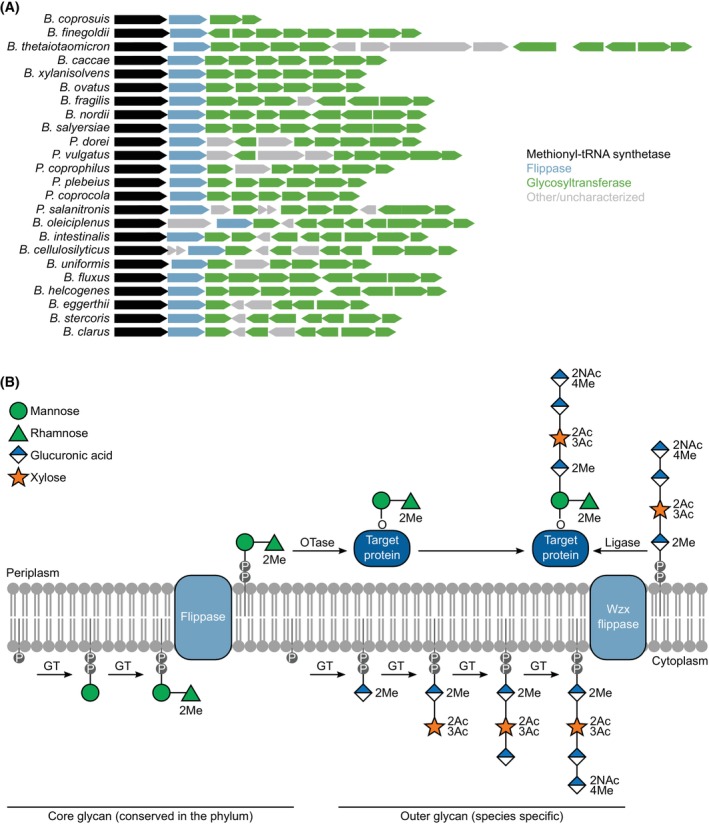
Biosynthesis of Bacteroidota *O‐*glycans. (A) Enzymatic machinery required for the synthesis of the *O‐*glycans is clustered in a genetic locus of similar structure across species, but shows diversity in the genes that are present. Proposed function is based on structural homology to other proteins in UniProt using Foldseek [34]. (B) Proposed model for the biosynthetic steps as previously described [22] and here exemplified for the species *Flavobacterium columnare* . Two separate glycans are synthesized on Und‐P lipid carriers in the cytoplasm by glycosyltransferases (GTs). The GTs that add specific sugar monomers are mostly uncharacterized. The core glycan (conserved across the phylum), and the outer glycan (species‐specific) are proposed to be synthesized separately. Both glycans are flipped to the periplasm by flippases, and the core glycan is thought to be attached to the target protein by an oligosaccharyl transferase (OTase). A ligase then adds the outer glycan to the core glycan. Me: *O*‐methyl; Ac: *O*‐acetyl; NAc: *N*‐acetyl.

The biosynthetic pathway of these glycans was first described in *B. fragilis*, in which a genomic region containing a flippase and multiple glycosyltransferases was identified and termed locus of fragilis glycosylation (LFG) [9]. Homologous loci in other species of the *Bacteroides* genus were later identified (some of these species are now considered a separate genus) [22]. These loci share a common genetic organization, starting with a methionyl‐tRNA synthetase (*metG*) followed by a *wzx* flippase and ending with two glycosyltransferases that are homologous across all species (Fig. 3A). Sandwiched between these genes is a series of other proteins that vary from species to species, with a large proportion of them predicted to be glycosyltransferases based on sequence and structural homology. These glycosyltransferases are largely uncharacterized, and only for a few examples do we know which monosaccharides they add to the glycan [23, 24, 29, 30, 32, 33]. The deletion of this locus (from the wzx flippase to the last glycosyltransferase) abolishes most of the glycan, leaving only the hexose and deoxyhexose that are common to most species characterized to date (Fig. 2C). Thus, a model has been proposed in which the glycan is synthesized in two independent steps (Fig. 3B): a core disaccharide that is more conserved and for which the machinery is encoded somewhere else in the genome; and an outer glycan that is highly variable and encoded by the genes in the LFG [22]. In homology to the OTase‐dependent *O*‐glycosylation pathway, it is thought that each glycan is assembled from nucleotide‐activated monosaccharides on the cytosolic side of the inner membrane prior to being flipped to the periplasm and transferred to the protein by an OTase for the core glycan and by a ligase for the outer glycan. The flippase for the core glycan, and the OTase and ligase remain undiscovered. The final *O*‐glycans are commonly modified with acetyl‐ and methyl‐groups, and in rare cases with phosphate‐, acetamidino‐, amino‐, glyceroyl‐, glycero‐, and/or sugar alcohol groups (Fig. 2C). It is currently unknown whether these modifications are already present on the nucleotide‐activated monosaccharides or whether they are introduced after the glycan assembly.

## Future outlook

Multiple aspects of these *O‐*glycans warrant further investigation, such as their function, the overall diversity of glycan structures, and their complete biosynthetic pathway.

### Function of Bacteroidota‐specific *O‐*glycosylation is still not completely understood

The function of these *O‐*glycans is currently largely unknown. Deletion of the outer glycan leads to a decrease in the number of viable *B. fragilis* cells at all growth stages [9]. This could imply that the glycans increase protein stability by either preventing denaturation or proteolytic degradation. These can be studied at the proteome level by measuring protein thermal [35, 36] or proteolytic stabilities [37, 38], so it will be interesting to use such approaches in this context.

It is also possible that these glycans are important for interactions with the host, since the outer glycan deletion mutant can colonize a mouse gut, but it has a fitness disadvantage when competed with the wild‐type strain [9]. In addition, truncations of the glycan in *T. forsythia* lead to higher Th17‐mediated neutrophil infiltration in the gingival tissue [26, 30, 32] and reduced levels of biofilm [39, 40]. The sugar chains of OmpA‐like protein of the same species interact with multiple selectins and siglecs [41], and the pseudaminic acid and legionaminic acid present in *T. forsythia* glycans might serve as molecular mimics of eukaryotic sialic acids to evade host immune response [30]. *O*‐glycosylated lipoproteins have also been shown to be present on outer membrane vesicles (OMVs), which have been suggested to redirect the immune system response away from the bacterial cell [42].

However, not all species of the Bacteroidota phylum are associated with a host. For example, some *Flavobacteria* species occupy marine and soil habitats. Together with the fact that the machinery responsible for the outer glycan synthesis seems conserved in most strains of the same species [22], this suggests that there is selective pressure for members of the same species to modify their proteins with the same glycan structure, which could imply that they are used for self‐recognition. Further studies are needed to identify specific interactions of the glycans with other biomolecules.

### Full extent of structural diversity remains to be explored

Our current view of the chemical diversity of these glycans is limited to a small number of species in restricted families (Fig. 2B), with only a few of them being fully characterized in terms of the exact monosaccharide composition and linkage types (Fig. 2C). Expanding this characterization to a larger number of species of the Bacteroidota phylum might reveal more bacterial‐specific monosaccharides and modifications. Currently, the only way to achieve this is by purification of the glycan and structure determination by nuclear magnetic resonance. A broader analysis in other species might expedite the characterization directly from mass spectrometry data, since specific monosaccharides and linkage types have been shown to result in predictable fragment ion intensities [43].

Furthermore, it might be interesting to expand this analysis to the sister phylum of Fibrobacterota, which shows a similar enrichment of conservation of the glycosylation motif and shares a common evolutionary root (Fig. 2A). This would allow pinpointing the origin of this glycosylation system.

### Full biosynthetic pathway is not characterized

The glycosyltransferases responsible for synthesizing the outer glycan appear located in a single locus in some *Bacteroides* and related genera (the LFG). Yet, it seems that this locus is not conserved across the whole Bacteroidota phylum. For example, in *P. gingivalis*, homologs of *B. fragilis* LFG proteins do not appear to be located closely together in the genome [24, 29]. With a few exceptions [23, 24, 29, 30, 32, 33], the specific reactions that each glycosyltransferase in this locus catalyzes are also largely unknown. Detailed molecular biology and biochemistry work deleting or purifying each of the components is necessary.

In addition, the machinery responsible for the synthesis of the core glycan that is shared across species has not been identified (Fig. 3B). This pathway resembles the eukaryotic *O‐*mannosylation system [44] and thus looking for homologs of that system in these species might offer insights into the proteins involved.

## Conclusion

While substantial progress has been made to characterize this glycosylation system, studying it in more detail could open up strategies for modulating the composition of the gut microbiome. Members of the Bacteroidota phylum are generally considered key species for a healthy microbiome but have sometimes been associated with disease [45]. The inhibition of specific glycosyltransferases to change the outer glycan might alter the fitness of specific bacteria and thus enable targeting specific species. Another strategy could be to use genetically engineered strains with specific glycans that can occupy a unique niche within the gut. Finally, these glycans could be potential targets for OMV‐targeted antibodies and vaccine development for systemic diseases associated with Bacteroidota and their OMVs [42].

## Conflict of interest

The authors declare no conflict of interest.

## Author contributions

All authors (L.H., D.S., and A.M.) contributed to the writing of the manuscript. A.M. supervised the work.

## Supporting information


**Fig. S1.** Glycosylated amino acid motif is similar across species.
