# Supplementary material for: Protein O‐glycosylation in the Bacteroidota phylum
Source: FEBS Open Bio. 2025 Apr 15;16(2):243–51. doi: 10.1002/2211-5463.70041 (PMC12871550; doi:10.1002/2211-5463.70041)
Supplement: Supplementary file 1 — Fig. S1. Glycosylated amino acid motif is similar across species. [file FEB4-16-243-s001.pdf]

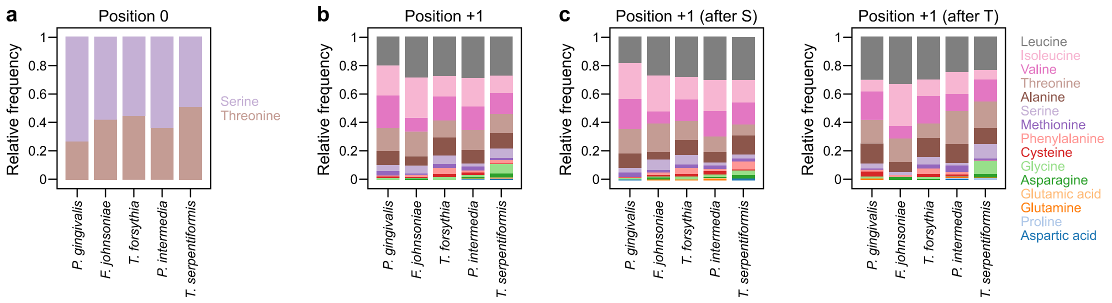

Supplementary Figure 1. Glycosylated amino acid motif is similar across species. (a) Distribution of species preference of serine or threonine for the amino acid that gets glycosylated. Based on the same data used in **Figure 1a**. **(b)** Distribution of species preference for the amino acid that follows the residue that gets glycosylated. **(c)** Same as in **b**, but depending on if the amino acid that gets glycosylated is serine or threonine.
